# Production, Storage Stability, and Susceptibility Testing of Reuterin and Its Impact on the Murine Fecal Microbiome and Volatile Organic Compound Profile

**DOI:** 10.3389/fmicb.2021.699858

**Published:** 2021-07-30

**Authors:** Christoph Castellani, Beate Obermüller, Bernhard Kienesberger, Georg Singer, Clemens Peterbauer, Reingard Grabherr, Sigrid Mayrhofer, Ingeborg Klymiuk, Angela Horvath, Vanessa Stadlbauer, Hannes Russmayer, Wolfram Miekisch, Patricia Fuchs, Holger Till, Stefan Heinl

**Affiliations:** ^1^Department of Paediatric and Adolescent Surgery, Medical University of Graz, Graz, Austria; ^2^Department of Food Science and Technology, University of Natural Resources and Life Sciences, Vienna, Austria; ^3^Department of Biotechnology, University of Natural Resources and Life Sciences, Vienna, Austria; ^4^Core Facility of Molecular Biology, Medical University of Graz, Graz, Austria; ^5^Department of Cell Biology, Histology and Embryology, Gottfried Schatz Research Center, Medical University of Graz, Graz, Austria; ^6^Division of Gastroenterology and Hepatology, Department of Internal Medicine, Medical University of Graz, Graz, Austria; ^7^Center of Biomarker Research in Medicine (CBmed), Graz, Austria; ^8^CD Laboratory for Biotechnology of Glycerol, Vienna, Austria; ^9^Department of Anesthesiology and Intensive Care, Experimental Research Center, University of Rostock, Rostock, Germany

**Keywords:** reuterin, 3-hydroxypropionaldehyde, microbiome, postbiotics, volatile organic compound, antimicrobial activity

## Abstract

**Background:** Probiotics are generally considered as safe, but infections may rarely occur in vulnerable patients. Alternatives to live microorganisms to manage dysbiosis may be of interest in these patients. Reuterin is a complex component system exhibiting broad spectrum antimicrobial activity and a possible candidate substance in these cases.

**Methods:** Reuterin supernatant was cultured from *Lentilactobacillus diolivorans* in a bioreactor in a two-step process. Storage stability at −20°C and effect of repeated freeze-thaw cycles were assessed by high performance liquid chromatography (HPLC). Antimicrobial activity was tested against *Clostridium difficile*, *Listeria monocytogenes*, *Escherichia coli*, *Enterococcus faecium*, *Staphylococcus (S.) aureus*, *Staphylococcus epidermidis*, *Streptococcus (S.) agalactiae*, *Propionibacterium acnes*, and *Pseudomonas aeruginosae*. Male BALBc mice were gavage fed with reuterin supernatant (*n* = 10) or culture medium (*n* = 10). Fecal volatile organic compounds (VOC) were assessed by gas chromatography mass spectroscopy; the microbiome was examined by 16S rRNA gene sequencing.

**Results:** The supernatant contained 13.4 g/L reuterin (3-hydroxypropionaldehyde; 3-HPA). 3-HPA content remained stable at −20°C for 35 days followed by a slow decrease of its concentration. Repeated freezing/thawing caused a slow 3-HPA decrease. Antimicrobial activity was encountered against *S. aureus*, *S. epidermidis*, and *S. agalactiae*. Microbiome analysis showed no differences in alpha and beta diversity markers. Linear discriminant effect size (LEfSe) analysis identified *Lachnospiraceae_bacterium_COE1* and *Ruminoclostridium_5_uncultured_Clostridiales_ bacterium* (in the reuterin medium group) and *Desulfovibrio_uncultured_ bacterium*, *Candidatus Arthromitus*, *Ruminococcae_NK4A214_group*, and *Eubacterium_xylanophilum_group* (in the reuterin group) as markers for group differentiation. VOC analysis showed a significant decrease of heptane and increase of 3-methylbutanal in the reuterin group.

**Conclusion:** The supernatant produced in this study contained acceptable amounts of 3-HPA remaining stable for 35 days at −20°C and exhibiting an antimicrobial effect against *S. aureus*, *S. agalactiae*, and *S. epidermidis*. Under *in vivo* conditions, the reuterin supernatant caused alterations of the fecal microbiome. In the fecal, VOC analysis decreased heptane and increased 3-methylbutanal were encountered. These findings suggest the high potential of the reuterin system to influence the intestinal microbiome in health and disease, which needs to be examined in detail in future projects.

## Introduction

The importance of the intestinal microbiome has gained wide scientific interest in health and disease. Intestinal bacteria have, among others, been associated with the fermentation of short-chained fatty acids from non-digestible oligosaccharides, synthesis of secondary and tertiary from primary bile acids and modulation of the intestinal immune system (Louis et al., 2014).

The composition of the intestinal microbiome is susceptible to nutritional changes, medication or chronic diseases such as tumor-associated cachexia, inflammatory bowel disease, or type two diabetes. Therefore, the modification of the intestinal bacterial composition toward a “healthier” microbiome has become attractive as possible therapeutic or supportive therapy approach. Apart from dietary modifications, this could be achieved by nutritional supplementation with pre-, pro-, or synbiotics. Probiotics are generally considered as safe. However, in vulnerable patient cohorts infections with probiotics may occur. Therefore, alternatives to live microorganisms to manage dysbiosis are of interest (Redman et al., 2014). In these cases, a helpful alternative in the treatment of intestinal dysbiosis is needed.

Some intestinal bacteria are capable of synthetization of specific substances inhibiting the growth or inactivating other competitive strains in order to defend their own habitat. This bacterial defensive strategy is based on different classes of antimicrobial compounds. Some of these like reutericin are short, *ribosomally synthesized*, post-translationally modified peptides termed bacteriocins (Ganzle et al., 2000; Ganzle, 2004). Other antimicrobial metabolites like acetic acid, lactic acid, or reuterin are small organic compounds (De Weirdt et al., 2012; Montiel et al., 2014). Due to their antimicrobial effects, these bacteriocins and antimicrobial metabolites may be attractive alternatives to treat dysbiosis for instance in immunocompromised patients.

The accumulation of reuterin (3-hydroxypropionaldehyde, 3-HPA) was firstly discovered in a medium containing glycerol in *Limosilactobacillus reuteri* (former *Lactobacillus reuteri*; Talarico et al., 1988; Vollenweider et al., 2003). Its antimicrobial activity was firstly described by Axelsson et al. (1989). 3-HPA is an intermediate in the metabolism of glycerol to 1,3-propanediol (1,3-PDO) and is formed by the dehydration of glycerol by many species belonging to genera such as *Klebsiella*, *Lactobacillus*, *Enterobacter*, *Citrobacter*, *Clostridium*, or *Eubacterium* (Vollenweider et al., 2003; Engels et al., 2016). While in most cases 3-HPA is immediately reduced to 1,3-PDO, *L. reuteri* and *Lentilactobacillus diolivorans* (among others) are able to secrete 3-HPA in low glucose environments (Engels et al., 2016). Excreted into an aqueous solution, the monomer 3-HPA undergoes a reversible dimerization and hydration, forming an equilibrium of 3-HPA (HPA-monomer), its hydrate (1,1,3-trihydroxypropane/1,1,3-propanetriol) and its dimer (2-(2-hydroxyethyl)-4-hydroxy-1,3-dioxane; Vollenweider et al., 2003). This complex mixture is known as HPA- or reuterin-system (Vollenweider et al., 2003). Since 3-HPA may undergo a spontaneous dehydration in aqueous solution, it was recently proposed to include the resulting molecule acrolein in the definition of reuterin (Engels et al., 2016). Although, the HPA-dimer was patented in 1988 under the name reuterin because it was held responsible for the antibiotic effect of the system (Vollenweider et al., 2003), the name reuterin is now more commonly used as a synonym for the system itself (Vollenweider and Lacroix, 2004).

Interestingly, 3-HPA concentrations of 15–30 μg per ml were shown to inhibit the growth of Gram-negative and Gram-positive bacteria, yeasts, fungi, and protozoa *in vitro* (Casas and Dobrogosz, 2000). The probiotic effect of lactobacilli has been tested in a variety of *in vivo* experiments yielding beneficial results in various diseases (Yadav et al., 2020). However, the majority of these studies have focused on the effects of supplementation of bacteria as probiotic and not on the effect of the reuterin system.

The aims of this study were (1) to synthesize the purest possible 3-HPA solution without vitamin B12 as co-enzyme, (2) to test the storage stability of this system, (3) to examine its *in vitro* antimicrobial activity, and (4) to examine its *in vivo* effect on the fecal microbiome and volatile organic compound (VOC) profile in a murine model.

## Materials and Methods

### 3-HPA Production

After a preliminary screening of several in-house *L. reuteri* and *L. diolivorans* strains regarding their ability to produce high amounts of 3-HPA in shaking flask experiments, the most efficient strain, *L. diolivorans* LMG 19668, was chosen as production strain. 3-HPA was biosynthetically produced using a two-step process according to Lindlbauer et al. (2017) with a few modifications. Thus, glycerol-containing water without vitamin B12 was used as fermentation medium in order to avoid substances, which could affect the animal experiments. A preculture was prepared by inoculating 40 ml MRS broth, 10 g/L peptone from casein (Merck), 10 g/L meat extract (VWR, Radnor, Pennsylvania), 5 g/L yeast extract (Fluka), 20 g/L glucose (Sigma Aldrich), 2 g/L dipotassium hydrogen phosphate (Sigma Aldrich), 1 ml/L Tween 80 (Sigma Aldrich), 2 g/L ammonium citrate tribasic (Sigma Aldrich), 5 g/L sodium acetate (Sigma Aldrich), 0,2 g/L magnesium sulfate (Merck), and 0,05 g/L manganese sulfate (Sigma Aldrich; pH 5.7) with 1 ml cryostock of the strain *L. diolivorans* LMG 19668 (Lindlbauer et al., 2017), followed by an overnight incubation at 30°C and 150 rpm in an anaerobic atmosphere. An aliquot of the preculture was used to obtain an initial optical density (OD) of 0.1 (absorbance unit) at 600 nm in 800 ml MRS broth (pH 5.7) supplemented with 5 mg/L vitamin B12 (Sigma Aldrich), 5.17 g/L 1,2-PDO (Sigma Aldrich), and additional 10 g/L glucose. This growth medium was previously added into one reactor of the Eppendorf parallel fermentation system (Eppendorf) with four parallel bioreactors. To avoid foam formation, 5 ml of a 10% Struktol® SB2121 solution (Schill Seilbacher, Hamburg, Germany) was also added. The inoculated medium was incubated under agitation (400 rpm) at 30°C. The anaerobic condition and the pH of 5.7 were kept constant during the incubation by gassing with N_2_ (2 sL/min) and automatic addition of NH_3_ (12.5%). Growth and thus biomass production were monitored by measuring the OD at 600 nm (OD_600_) in a photometer (WPA Biowave, CO8000 Cell Density Meter; WPA, Cambridge, United Kingdom) after diluting the sample 10 times in water. Samples were taken at the beginning and at the end of biomass generation as well as at regular intervals (approximately 60 min) during the entire biotransformation period. At the end of the exponential phase, when NH_3_ consumption was stopped and CO_2_ generation was reduced, the cells were harvested by centrifugation (4,248 g, 8 min, 20°C).

The obtained cell pellet was washed in 200 ml water and finally resuspended in 400 ml of a 2% glycerol (Carl Roth GmbH) solution. The pH of the solution was adjusted to and maintained at 7 using NH_3_ (12.5%). The biotransformation of glycerol took place in an Eppendorf parallel fermentation system at 30°C, 400 rpm agitation and 2 sL/min N_2_-gassing. For biotransformation, the concentrations of the extracellular metabolites glycerol and 3-HPA were determined by high performance liquid chromatography (HPLC) analysis (Shimadzu, Korneuburg, Austria) with an Aminex HPX-87H column (300 mm × 7.8 mm; Bio-rad Laboratories, Feldkirchen, Germany) equipped with a Micro-Guard Cation H Cartridge (30 mm × 4.6 mm; Bio-rad Laboratories, Feldkirchen, Germany) at defined time points (00:00; 00:50; 01:45; 02:50, and 03:20 h) as reported in the literature (Pflugl et al., 2012). The column was operated at 60°C, 0.6 ml/min flow rate and 0.004 M H_2_SO_4_ as mobile phase. The process was completed when the amount of 3-HPA stopped increasing. The solution was then centrifuged (4,248 g, 8 min, 20°C), filter-sterilized, portioned into 50 ml screw cap tubes and stored at −20°C until provision. This solution will be referred to as reuterin supernatant in the following.

### Storage Stability Testing

To test storage stability of 3-HPA, sterile-filtrated reuterin supernatant was distributed to 1.5 ml Eppendorf vials (0.9 ml each vial) and stored at −20°C. Measurements were conducted at different time points throughout 1 year. Before each measurement, two tubes of the frozen supernatant were thawed at 4°C for 1 h. The concentrations of 3-HPA and glycerol were determined by HPLC analysis as described above.

To determine the stability of 3-HPA in the reuterin supernatant after several freeze-thaw cycles sterile-filtrated supernatant was portioned into 15 and 50 ml screw cap tubes, respectively. Both tubes were stored at −20°C, thawed to 4°C and tested weekly for the presence of 3-HPA and glycerol by HPLC over a period of 28 days.

### Antibacterial Activity Testing

Bacteria for resistance testing were obtained from Aurosan GmbH, Essen, Germany. *Clostridium difficile* (ATCC 700057), *Listeria monocytogenes* (ATCC 15313), *Escherichia coli* (ATCC 25922), *Enterococcus faecium* (ATCC 27270), *Staphylococcus (S.) aureus* (ATCC 29213), *Staphylococcus (S.) epidermidis* (ATCC 12228), *Streptococcus (S.) agalactiae* (ATCC 13813), *Pseudomonas (P.) aeruginosa* (ATCC 27853), and *Propionibacterium (P.) acnes* (ATCC 6919) were chosen for bacterial resistance testing. After thawing, bacteria were pre-cultured as broth cultures. Aerobic bacteria were cultivated for 24 h at 37°C and 360 rpm and anaerobic bacteria for 48 h at 37°C and 360 rpm. Of each pre-culture, 300 μl were then spread on 15 cm diameter plates with either tryptic soy agar (30 g/L TSB, Fluka Analytical, 15 g/L Agar-Agar, Kobe I, Carl Roth) for *E. coli*, *S. aureus*, and *P. aeruginosa*, TSA with 5% freshly harvested sheep blood for *C. difficile*, *S. agalactiae*, and *P. acnes*, brain heart infusion agar (Brain-Heart-Infusion, 52 g/L, Carl Roth; 15 g/L Agar-Agar Kobe I, Carl Roth) for *E. faecium* and *L. monocytogenes* or nutrient agar (1 g/L beef extract powder, Sigma-Aldrich; 5 g/L peptone, Sigma-Aldrich; 5 g/L NaCl and 15 or 50 g/L Agar-Agar) for *S. epidermidis*. Anaerobic bacteria (*C. difficile* and *P. acnes*) were cultured for 48 h in boxes with oxid Anaerogen 2.5 L (Thermo Fisher Scientific). The remaining aerobic bacteria grew for 24 h until a dense bacterial lawn was achieved.

Each microorganism was cultured on five different plates. Using a stencil, 10 disks for resistance testing (BD Sensi-Disc^tm^, Becton, Dickinson and Company, Franklin Lakes, New Jersey, United States) were placed on each culture plate. For resistance testing, reuterin supernatant was thawed to 4°C. Each disk was either treated with respectively 20 μl of pure supernatant, a 1:2 or 1:4 dilution of the supernatant, cooked supernatant (100°C for 30 min at 10,000 rpm), buffered supernatant (to pH 7 with 1 n NaOH), supernatant mixed with 1 n HCl (1:1) or supernatant treated with 1 mg/ml Proteinase K (Carl Roth). Biotransformation medium (a 6.9 g/L glycerol solution), buffered biotransformation medium (to pH 7 with 1 n NaOH) as negative control and antibiotic positive control. Either vancomycin (Vancomycin Hikma® 500 mg, Hikma Pharma; 0.03 mg/disk); for *C. difficile*, *S. agalactiae*, *E. faecium*, *S. epidermidis*, *L. monocytogenes*, and *S. aureus* or Piperacillin/Tacobactam (PIPeracillin/TAZobactam Kabi 4 g/0.5 g, Fresenius Kabi; 0.1 mg/disk) for *E. coli*, *P. aeruginosa*, and *P. acnes* were used as positive controls. Plates were then incubated at 37°C for 24 h in case of aerobic and for 48 h in case of anaerobic bacteria. Thereafter, plates were photographed and inhibition zones were determined with ImageJ 2.0.0-rc-69/1.52p (ImageJ open source image processing software).^1^

### *In vivo* Application

For *in vivo* application, a supernatant containing 13.4 g/L 3-HPA and 6.9 g/L glycerol was used. A biotransformation medium containing 6.9 g/L glycerol served as control. The supernatant was thawed once for aliquoting and dilution with sterile water (1:10 or 1:20) to 1.5 ml Eppendorf vials and then stored at −20°C. BALBc mice (*n* = 20) were obtained at an age of 7 weeks from the Center for Biomedical Research of the Medical University of Vienna, Austria as one patch of littermates for microbiome testing. After delivery and an acclimatization period of 2 weeks, mice were split forming two equal groups (*n* = 10 each) with equal body weight distribution. Animal experiments were approved by the veterinary board (BMBWF-66.010/0153-V/3b/2019). Mice were kept single-housed in individually ventilated cages under specific pathogen free conditions, a 12 h light-dark cycle and an allowed free access to chow and water at all times. After acclimatization, mice underwent a daily gavage of 400 μl with reuterin supernatant (2 weeks 1:20 solution equaling 0.268 g 3-HPA and 0.138 g glycerol per day) to accustom to the substance followed by 1:10 (equaling 0.536 g 3-HPA and 0.276 g glycerol per day) for 4 weeks in the intervention (reuterin) group. In the control (reuterin medium) group, biotransformation medium was diluted 1:10 or 1:20 similar to the intervention group. Mice received a daily gavage of 400 μl with 1:20 solution (equaling 0.138 g glycerol per day) for 2 weeks followed by 1:10 solution (equaling 0.276 g glycerol per day) for 4 weeks. Mice were euthanized after 6 weeks of treatment. Two stool samples were collected at euthanasia. One was stored at −80°C until 16S based microbiome analysis. The other sample was immediately sent for VOC analysis.

### 16S rRNA Gene Based Microbiome Analysis

For total DNA isolation, fecal samples were isolated with the Magna Pure LC DNA III Isolation Kit (Bacteria, Fungi; Roche) according to published protocols (Klymiuk et al., 2017). Briefly, one stool pellet was mixed with 500 μl PBS 10x and 250 μl bacterial lysis buffer. Samples were homogenized and bead beaten in Magna Lyzer Green bead Tubes (Roche) in a Magna Lyzer instrument (Roche) at 6,500 rpm for 30 s two times. Followed by enzymatic lysis with 25 μl lysozyme (100 ng/ml, 37°C for 30 min) and 43,4 μl proteinase K (20 mg/ml, 65°C for 1 h) samples were heat inactivated at 95°C for 10 min and total DNA was purified in a MagnaPure LC instrument according to manufacturer’s instructions. Total DNA was eluted in 100 μl elution buffer and stored at −20°C until analysis. For 16S PCR, 2 μl of total DNA were used as template in a 25 μl PCR reaction with the FastStart™ High Fidelity PCR-System (Sigma Aldrich) according to manufacturer’s instructions and the target specific primers 515F (5'-GTGYCAGCMGCCGCGGTAA-3') and 806R (5'-GGACTACNVGGGTWTCTAAT-3') for 30 cycles in triplicates. Triplicates were pooled, normalized, indexed, and purified according to published protocols (Klymiuk et al., 2017). The final pool was sequenced on an Illumina MiSeq desktop sequencer at 9 pM and v 3,600 cycles chemistry. FastQ raw files were used for data analysis.

A total of 2.479.083 MiSeq paired end FASTQ reads were used further analysis. The DADA2 pipeline for modeling and correcting Illumina-sequenced amplicon errors for quality-filtering (Callahan et al., 2016) was used with standard settings for denoising, dereplicating, merging, and check for chimeras as implemented in QIIME2 2018.4 microbiome bioinformatics platform (Bolyen et al., 2019). QIIME2 was integrated in an own non-public instance of Galaxy (MedBioNode; Afgan et al., 2018).^2^ Taxonomic assignment of the DADA2 representative sequences was provided with the QIIME2 sklearn-based classifier against SILVA rRNA database release 132 at 99% identity (Quast et al., 2013). Absolute counts from OTU table on genus level were used to assess abundance changes. To interpret and compare taxonomic information 16S rRNA data was transferred to the Calypso online software (Calypso 8.84®, accessible through http://cgenome.net/wiki/index.php/Calypso; Zakrzewski et al., 2017). Samples were rarefied to a read depth of 20,220. Alpha diversity was calculated using Chao1 estimator, Inversed Simpson, and Shannon index. Beta diversity was examined using a redundancy analysis (RDA) and colored principal component analysis plots (PCoA) based on Bray-Curtis dissimilarity score. Values of *p* were adjusted for multiple testing by FDR. The identification of discriminating taxa between the groups was performed with a linear discriminant effect size (LEfSe) analysis. Differentially abundant taxa identified by LEfSe analysis were considered relevant if the differences between groups could be verified by ANOVA (*p* < 0.1).

### Fecal Volatile Organic Compound Profile

At euthanasia, fecal samples were harvested to glass vials (Gerstel GmbH, Germany) and stored at 6°C. Room air samples were collected at the same time points to correct for possible contamination. All samples were immediately sent to the partner *via* overnight express for gas chromatography/mass spectroscopy. VOC analysis was performed in the headspace of stool samples as previously reported (Miekisch et al., 2014; Bergmann et al., 2015; Obermuller et al., 2020). VOCs were pre-concentrated with a commercially available solid phase micro extraction (SPME) fiber (carboxen/polymethylsiloxane, Sulpeco, Bellefonte, PA, United States). An Agilent 7890 A gas chromatograph (GC) coupled to an Agilent 5975 C inert XL mass selective detector (MSD) was used to separate and identify the VOCs desorbed from the SPME device. Detected marker substances were identified from a mass spectral library (National Institute of Standards and Technology 2005; NIST 2005, Gatesburg, PA, United States) and by retention time matching. In case the median of the room air samples exceeded 30% of the median of the stool samples a possible contamination was recorded and the substance was excluded from further analysis. The responses of a selected m/q ratio at a defined retention time for each substance were recorded, integrated, and used for group comparison.

### Statistics

Data were managed with Microsoft Excel 2016®. For statistical analysis, data were transferred to SPSS 26.0®. Graphical work-up was performed with GraphPad Prism 9®. Metric data are displayed as median and interquartile range (IQR). A Mann-Whitney-U-Test was used to determine group differences. A Spearman-Rho analysis was performed to search for correlations between relative bacterial abundances and VOC substances. Values of *p* < 0.05 were considered statistically significant.

## Results

### Reuterin Production

Under the culture conditions described above a supernatant containing a maximum of 13.4 g/L 3-HPA and 6.8 g/L glycerol could be obtained (Table 1). Supplement 1 shows an exemplary HPLC curve of the supernatant produced in this study.

**Table 1:** 3-hydroxypropionaldehyde (3-HPA) and glycerol content in the supernatant at different time points in a duplicate approach.

Bioreactor 1/2	Time (hh:mm)				
	00:00	00:50	01:45	02:50	03:20
3-HPA (g/L)	1.1/0.7	7.8/6.6	10.4/9.6	10.3/9.4	13.4/11.9^*^
Glycerol (g/L)	23.2/23.48	15.5/15.63	9.3/10.9	9.0/10.6	6.8/8.3

* The supernatant with the higher 3-HPA value was used for the further experiments.

### Storage Stability Testing

After a small initial drop, the 3-HPA concentration remained stable until day 35 at −20°C followed by a slow decrease in the following measurements (Table 2). Freezing and thawing of the supernatant lead to a slow decrease of 3-HPA with every cycle. This decrease was more pronounced in the 50 ml compared to the 15 ml storage vials (Table 3).

**Table 2:** Average content of 3-HPA and glycerol in the supernatant (in % of the initial value) of the two samples from bioreactor 1 examined at different time points after storage at −20°C.

Day	3-HPA (g/L)	Glycerol (g/L)
0	100%	100%
7	94%	101%
9	94%	101%
12	94%	101%
14	94%	101%
35	94%	101%
63	68%	101%
211	72%	123%
244	70%	123%
302	69%	124%
342	66%	123%
369	66%	122%

**Table 3:** Average 3-HPA and glycerol content in the supernatant (in % of the initial value) after freeze-thaw cycles performed weekly for 28 days in 15 and 50 ml vials.

Day	3-HPA (g/L)		Glycerol (g/L)	
	15 ml (%)	50 ml (%)	15 ml (%)	50 ml (%)
0	100	100	100	100
7	97	89	102	93
14	95	86	101	93
21	94	85	101	93
28	93	85	102	94

### *In vitro* Antibacterial Activity

**Figure 1:**
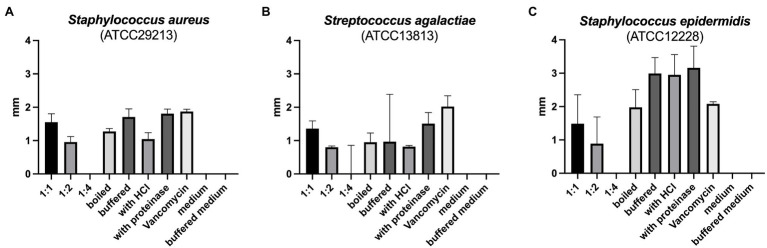
**(A–C)** Results of antimicrobial resistance testing. Bars represent the median diameter in mm, whiskers the interquartile range (IQR) of the five samples.

The reuterin supernatant investigated in this experiment exhibited antimicrobial activity against *S. aureus*, *S. epidermidis*, and *S. agalactiae* but not against the other bacteria tested (Figure 1). The antimicrobial activity decreased with increasing dilution of the supernatant. Neither boiling, nor buffering or proteinase K treatment had relevant influences on the antimicrobial activity. Similarly, treatment with HCl simulating exposure to the acidic gastric environment had no marked effect on the antimicrobial activity of the reuterin supernatant.

### *In vivo* Alterations of the Fecal Microbiome

**Figure 2:**
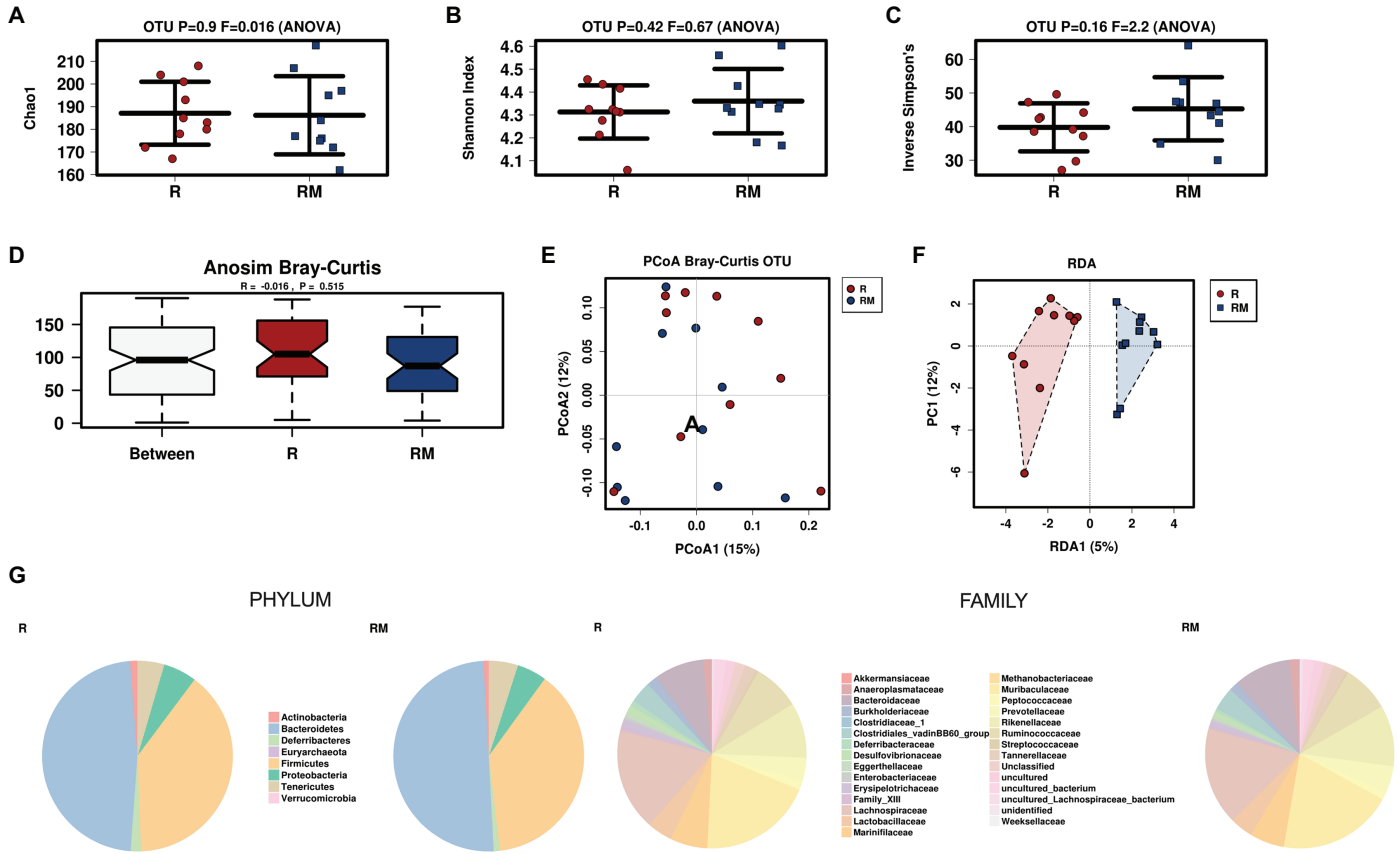
Alpha diversity markers **(A-C)**, beta diversity analysis **(D-F)**, and relative abundances of the two groups **(G)**. The redundancy analysis (RDA) revealed a value of *p* of 0.551. R, reuterin supernatant (reuterin group); RM, biotransformation medium (reuterin medium group).

**Figure 3:**
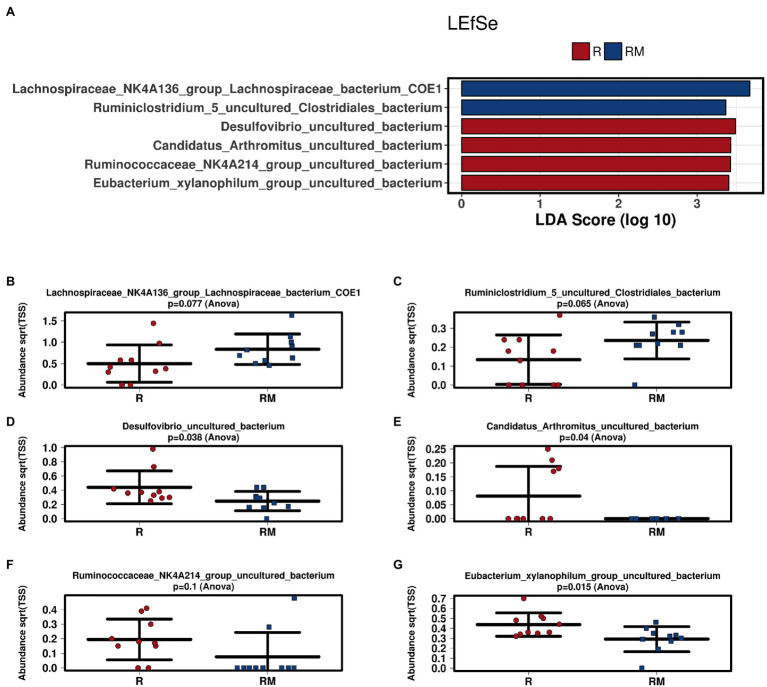
Linear discriminant effect size (LEfSe) analysis **(A)** and bar charts **(B–G)** of the two groups. R, reuterin supernatant (reuterin group); RM, biotransformation medium (reuterin medium group).

**Figure 4:**
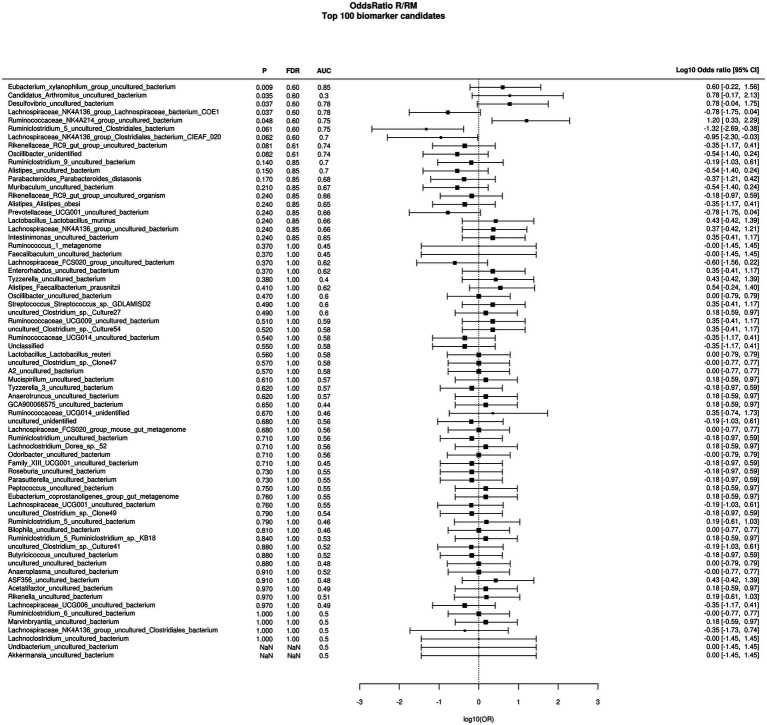
Odds ratio biomarker analysis of the two groups. R, reuterin supernatant (reuterin group); RM, biotransformation medium (reuterin medium group).

During *in vivo* application (in both groups), mice did not exhibit gastro-intestinal side effects (diarrhea, impaired feeding). The two murine groups did not differ in their body weight at delivery (reuterin median 25.2 g IQR 2.3; reuterin medium median 25.2 g IQR 2.0; *p* = 0.971) and euthanasia (reuterin median 29.1 g IQR 4.2; reuterin medium median 29.2 g IQR 1.8; *p* = 0.853). After a rarefication to 20,220 reads, alpha diversity indices (Chao1, Shannon and Inversed Simpson) were not significantly different between the two groups. Regarding beta diversity neither Anosim Bray Curtis nor PCoA Bray-Curtis or RDA showed significant differences between the groups (Figure 2). The LEfSe analysis identified *Lachnospiraceae_bacterium_COE1* and *Ruminoclostridium_5_uncultured_Clostridiales_ bacterium* (associated with the reuterin medium group), as well as *Desulfovibrio_uncultured_ bacterium*, *Candidatus Arthromitus*, *Ruminococcae_NK4A214_group*, and *Eubacterium_xylanophilum_group* (associated with the reuterin group) with a linear discrimination analysis (LDA) score > 3 as markers for group discrimination (Figure 3). Consequently, the same bacteria could also be identified for group discrimination in the biomarker analysis (Figure 4). The group comparison with ANOVA revealed a decrease of *Lachnospiraceae_bacterium_COE1* and *Ruminoclostridium_5_uncultured_Clostridiales_ bacterium* in the reuterin group. *Desulfovibrio_uncultured_ bacterium*, *Candidatus Arthromitus*, *Ruminococcae_NK4A214_group*, and *Eubacterium_xylanophilum_group* were increased in the animals treated with reuterin supernatant.

### *In vivo* Fecal VOC Profile

**Figure 5:**
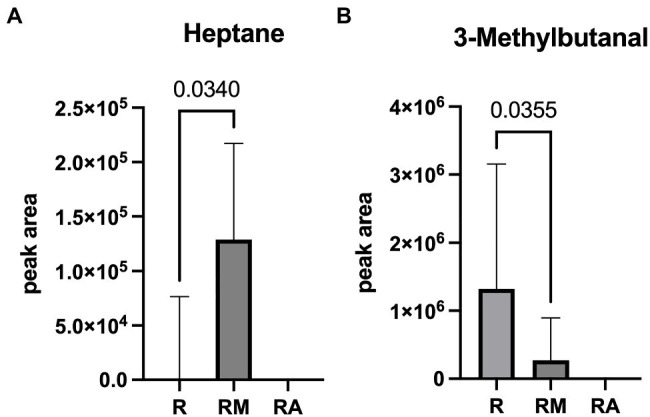
Heptane **(A)** and 3-Methylbutanal **(B)** as volatile organic compounds (VOCs) with significant group difference. R, reuterin supernatant (reuterin group); RM, biotransformation medium (reuterin medium group); RA, room air control.

A total of 42 different substances could be detected in the fecal samples. Alterations in four substances (propene, isopropyl alcohol, isoflurane, and propanol) were attributed to room air contamination leaving 38 for group comparison. A list of all detected substances is presented in Supplement 2. Heptane and 3-methylbutanal showed significant group differences (Figure 5). Reuterin supernatant treatment caused a significant decrease of heptane and a significant increase of 3-methylbutanal. There was no significant correlation between these substances and the relative abundance of fecal bacteria with significant group differences in the LEfSe analysis.

## Discussion

In this study, reuterin (3-HPA) with a relative storage stability of 35 days was synthesized. 3-HPA displayed *in vitro* antimicrobial activity against three aerobic Gram-positive cocci. Moreover, *in vivo* treatment with reuterin supernatant led to alterations of the fecal microbiome and VOCs.

Apart from lactobacilli, the natural production of 3-HPA has also been reported for species of the genera *Bacillus*, *Citrobacter*, *Clostridium*, *Enterobacter*, and *Klebsiella* (Lindlbauer et al., 2017). Lactobacilli, however, are one of the most frequent groups of prokaryotes used in industry because they commonly produce a large number of bioactive molecules (Mora-Villalobos et al., 2020). Previous studies have shown that in addition to *L. reuteri*, *L. brevis* (Schutz and Radler, 1984), *L. buchneri* (Schutz and Radler, 1984), *L. collinoides* (Sauvageot et al., 2000), *L. coryniformis* (Martin et al., 2005), *L. diolivorans* (Garai-Ibabe et al., 2008), *L. hilgardii* (Pasteris and Saad, 2009), and *L. pentous* (Bauer et al., 2010) can also metabolize glycerol to 1,3-PDO *via* 3-HPA. Normally, 3-HPA is an intracellular intermediate that does not accumulate but is reduced to 1,3-PDO, which is finally secreted into the extracellular space (Bauer et al., 2010). Only a few strains of *L. reuteri*, *L. coryniformis*, and *L. diolivorans* with the ability to extracellularly accumulate 3-HPA have been identified (Pasteris and Saad, 2009). The underlying mechanism, however, currently remains unknown.

Besides an appropriate bacterial strain, its concentration and age, the fermentation for bioconversion should also involve a suitable medium (Luthi-Peng et al., 2002; Lee and Paik, 2017). Experiments have shown that 3-HPA production largely depends on glycerol concentration and other ingredients of the medium (Luthi-Peng et al., 2002). Using complex growth media such as MRS supplemented with glycerol, high amounts of 3-HPA were produced by *L. reuteri* (Vollenweider et al., 2003). However, after purification the sample still has contained inadvertent by-products (Vollenweider et al., 2003). Therefore, a two-step process consisting of the generation of active biomass in a suitable growth medium (MRS medium) under optimal conditions, followed by a quick bioconversion with non-growing but metabolically active cells (resting cells) of *L. diolivorans* LMG 19668 in glycerol-containing water (biotransformation medium) was chosen for the production of 3-HPA in this project (Lindlbauer et al., 2017). This process has been described by Lindlbauer et al. (2017) to achieve the best results in terms of high productivity and suppression of by-product formation, since the metabolic pathways in growing cells are tightly balanced to prevent the accumulation of toxic 3-HPA. To avoid a further reduction of 3-HPA to 1,3-propanediol, biotransformation of glycerol to 3-HPA was carried out in the absence of glucose, as glycolysis would allow the formation of nicotinamide adenine dinucleotide hydrogen (NADH), which would promote this reduction (Pflugl et al., 2012). Another advantage is the final presence of 3-HPA in an aqueous solution instead of a complex medium (Krauter et al., 2012). In this set-up, a final 3-HPA concentration of 13.4 g/L was achieved with *L. diolivorans*. These results are comparable to reports of trials with *L. reuteri* ATCC 53608 reported in the literature (Luthi-Peng et al., 2002; Vollenweider et al., 2003; Doleyres et al., 2005).

According to the literature, 3-HPA is stable in water for at least 6 months at 4°C in both low and high concentrations (Luthi-Peng et al., 2002; Vollenweider et al., 2003). In addition, no changes in the composition of the HPA-system were measured over a period of 150 days at 4°C (Vollenweider et al., 2003). Since the stability of 3-HPA decreases with increasing temperature (Luthi-Peng et al., 2002), the opposite could be assumed for decreasing temperatures. Investigating the stability of 3-HPA in the supernatant of this study at −20°C, a slight decrease of the 3-HPA concentration could be observed at the beginning followed by a stable phase over 35 days and a further slow decrease throughout the following months. A possible reason for the discrepancy between the results of this investigation and those previously published in the literature may be found in the different applied methods for 3-HPA quantification. In this regard, it must be pointed out that methods other than HPLC were used for the quantification of 3-HPA by Luthi-Peng et al. (2002) and Vollenweider et al. (2003). Apart from the temperature, the stability of 3-HPA depends on sugar and free amino groups, since the functional groups of 3-HPA, the highly reactive hydroxyl and/or aldehyde group, may react with sulfhydryl, hydroxyl, carboxyl, and amino groups of biological molecules (Luthi-Peng et al., 2002; Vollenweider et al., 2003; Vollenweider and Lacroix, 2004; Doleyres et al., 2005; Cleusix et al., 2007). Consequently, these authors found that 3-HPA was more unstable in media other than water (Luthi-Peng et al., 2002). With long storage periods, the pH additionally had a significant effect on the stability of the main compounds of the HPA-system. Accordingly, the HPA-system was destroyed after 1 day or 7 days under strong acidic or alkaline conditions (Vollenweider et al., 2003).

Apart from the storage period, the influence of repeated freeze-thaw cycles on the 3-HPA content was also examined and a decrease in its concentration with every cycle was found. This decrease was found to be more pronounced in larger than in smaller vials. Therefore, factors such as the presence of oxygen or the contact surface could be speculated to influence the stability of 3-HPA. As a consequence, the 3-HPA supernatant was stored at −20°C and pH 7 for as little time as possible reducing freeze-thaw cycles as much as possible for the remaining *in vitro* and *in vivo* experiments of this study.

Regarding the antimicrobial effects, inhibitory activity of reuterin against Gram-positive and Gram-negative bacteria, yeasts, molds, and protozoa, including various food spoilers and pathogens, lactic acid bacteria used in food fermentations, and microorganisms that reside in the mammalian gastrointestinal tract has been reported (Engels et al., 2016). Given the complexity of the reuterin system, the question arises which molecule (3-HPA, HPA-dimer, or HPA-hydrate) is the active antimicrobial substance (Vollenweider and Lacroix, 2004). The antimicrobial activity of the reuterin system has been attributed to depletion of free thiol groups in glutathione, proteins, and enzymes resulting in an imbalance of the cellular redox status leading to bacterial cell death (Schaefer et al., 2010). Recent investigations could demonstrate, that acrolein seems to be the component of the system, which is mainly responsible for its antimicrobial activity (Engels et al., 2016; Asare et al., 2018, 2020).

Cleusix and coworkers have reported that a representative panel of human intestinal bacteria were generally sensitive to reuterin (Cleusix et al., 2007). Among the 19 non-*Lactobacillus* species tested, 15 have shown minimal inhibitory concentration values lower than that of *E. coli* K12, including *Bacteroides fragilis*, *Bacteroides thetaiotaomicron*, *Bacteroides vulgatus*, *Bifidobacterium adolescentis*, *Bifidobacterium bifidum*, *Bifidobacterium catenulatum*, *Bifidobacterium longum*, *Bifidobacterium longum* var. *infantis*, *Colinsella auerofaciens*, *Clostridium difficile*, *Enterococcus faecium*, *Eubacterium biforme*, *Eubacterium eligens*, *Listeria ivanovii*, and *Streptococcus salivarius* (Cleusix et al., 2007).

In this investigation, the reuterin supernatant produced as described above exhibited antimicrobial effects on the aerobic Gram-positive cocci *S. aureus*, *S. agalactiae*, and *S. epidermidis* but not on *C. difficile*, *E. faecium*, *L. monocytogenes*, *P. acnes*, and *P. aeruginosa*. While the influence of the reuterin system on some of these bacteria has not been described in the literature, the results for *E. faecium* and *C. difficile* contradict reports from other authors (Cleusix et al., 2007). However, the comparison of data from different studies is difficult due to various factors including the test method, the tested bacteria and the composition of the reuterin system. Limitations should also be considered when extrapolating *in vitro* reuterin sensitivity data to *in vivo* conditions such as the gastrointestinal tract (Cleusix et al., 2007). Thus, factors like nutrient competition and antagonistic or synergistic relationships between the intestinal microbiota can influence the inhibitory effects of reuterin (Cleusix et al., 2007). However, already low concentrations of reuterin in the intestine seem to be sufficient to alter bacterial growth (Cleusix et al., 2007).

Under *in vivo* conditions, the application of reuterin supernatant led to significant alterations of the murine fecal microbiome. To the best of our knowledge, the *in vivo* effect of reuterin on the intestinal/fecal microbiome has not been examined yet. Prior to the *in vivo* application, the influence of HCl and proteinase K on the reuterin supernatant was examined showing no marked decrease of its’ antimicrobial activity. Consequently, neither the acidic gastric milieu nor intestinal proteinases have an impact on the activity of the reuterin supernatant in the digestive tract (Soltani et al., 2021). Additionally, a recent publication by Soltani et al. (2021) revealed, that viability and membrane integrity of cells remained unaltered by reuterin up to a concentration of 1,080 mM. Furthermore, no hemolysis was observed in blood cells of rats exposed to 270 mM reuterin (Soltani et al., 2021).

Regarding microbiome analysis, no difference in alpha or beta diversity could be observed. Linear discriminant analysis effect size (LEfSe) was chosen for group comparison because it determines features potentially able to explain differences among conditions rather than the features that simply possess uneven distributions among classes (Segata et al., 2011). LDA was applied to estimate the effect size of each differentially abundant feature (Segata et al., 2011). All bacteria detected in the group comparison in this manuscript had LDA scores > 3 qualifying them for group discrimination. *Lachnospiraceae_bacterium_COE1* and *Ruminoclostridium_5_uncultured_Clostridiales_bacterium* were associated with the reuterin medium group. *Desulfovibrio_uncultured_bacterium*, *Candidatus Arthromitus*, *Ruminococcae_NK4A214_group*, and *Eubacterium_xylanophilum_group* were related to the reuterin group. In the ANOVA analysis of this study, a decrease of *Lachnospiraceae_bacterium_COE1* and *Ruminoclostridium_5_uncultured_Clostridiales_bacterium* as well as an increase of *Desulfovibrio_uncultured_bacterium*, *Candidatus Arthromitus*, *Ruminococcae_NK4A214_group*, and *Eubacterium_xylanophilum_group* could be demonstrated. To the best of our knowledge, there is currently no other available literature concerning the influence of reuterin on the murine fecal microbiome. Possible effects of theses alterations and their impact on disease have to be addressed in future studies.

Volatile organic compounds in the headspace of fecal samples are generated during metabolic processes within the intestine and are influenced by the intestinal epithelium, the microbiome and diet (Ahmed et al., 2013). Alterations of heptane have, among others, been described in cultures of human Simpson Golabi Behmel Syndrome adipocytes (Mochalski et al., 2019), in the supernatant of bacterial cultures associated with infectious bacterial activity (Qader et al., 2015), in breath samples of patients with bronchiolitis obliterans syndrome (Kuppers et al., 2018) or sleep apnea (Aoki et al., 2017). The VOC 3-methylbutanal has been detected in feces of patients with *Clostridium difficile* infection (Patel et al., 2019) and in bacterial cultures of *Staphylococcus aureus* and *Pseudomonas aeruginosa* (Filipiak et al., 2012). Furthermore, cultures of mesophilic lactobacilli (*Lactobacillus casei*, *Lactobacillus plantarum*, *Lactobacillus paracasei*, and *Lactobacillus helveticus* 209) were shown to produce 3-methylbutanal among other VOCs (Cuffia et al., 2020). Due to the multitude of intestinal bacteria and their metabolic processes, it is currently impossible to exclusively link specific VOCs to single bacteria. There was no statistically significant correlation between relative abundances of bacteria with significant group differences in the LEfSe analysis and fecal VOCs. Since the microbiome changes throughout the intestinal tract, it might be possible that the VOCs detected were produced upstream and then excreted with the feces. Because intestinal contents from other levels of the gastrointestinal tract were not harvested in this investigation, this question cannot be addressed at the moment.

In conclusion, the supernatant produced in this study contained acceptable amounts of 3-HPA remaining stable for 35 days at −20°C and exhibiting an antimicrobial effect against *S. aureus*, *S. agalactiae*, and *S. epidermidis*. Under *in vivo* conditions, the reuterin supernatant caused alterations of the fecal microbiome. In the fecal, VOC analysis decreased heptane and increased 3-methylbutanal were encountered. These findings suggest the high potential of the reuterin system to influence the intestinal microbiome in health and disease, which needs to be examined in detail in future projects.

## Data Availability Statement

The data presented in this study are deposited in the European Nucleotide Archive (https://www.ebi.ac.uk/ena/browser/home), accession number PRJEB45825.

## Ethics Statement

The animal study was reviewed and approved by Bundesministerium Bildung, Wissenschaft und Forschung BMBWF-V/3b Tierversuschswesen und Gentechnik, Minoritenplatz 5 1010 Vienna, Austria (Ref: BMBWF-66.010/0153-V/3b/2019).

## Author Contributions

CC wrote the manuscript. BO performed the animal experiments and the resistance testing and critically reviewed the manuscript. BK helped with the animal experiments, the resistance testing, and critically reviewed the manuscript. GS performed the statistical analysis and graphical workup and critically reviewed the manuscript. CP planned the reuterin production and critically reviewed the manuscript. RG supervised reuterin production and storage stability testing and critically reviewed the manuscript. SM produced the reuterin, performed the storage stability testing, and critically reviewed the manuscript. IK performed the microbiome analysis and critically reviewed the manuscript. AH helped with bacterial resistance testing and critically reviewed the manuscript. VS helped with the planning of the project and critically reviewed the manuscript. HR performed the HPLC analysis and critically reviewed the manuscript. WM and PF performed the VOC analysis and critically reviewed the manuscript. HT critically reviewed the manuscript. SH supervised reuterin production and statistics, helped to plan the entire study, and critically reviewed the manuscript. All authors contributed to the article and approved the submitted version.

## Conflict of Interest

The authors declare that the research was conducted in the absence of any commercial or financial relationships that could be construed as a potential conflict of interest.

## Publisher’s Note

All claims expressed in this article are solely those of the authors and do not necessarily represent those of their affiliated organizations, or those of the publisher, the editors and the reviewers. Any product that may be evaluated in this article, or claim that may be made by its manufacturer, is not guaranteed or endorsed by the publisher.

## Footnotes

^1^ http://imagej.net/Contributors

^2^ https://galaxy.medunigraz.at
