# Supplementary material for: Production, Storage Stability, and Susceptibility Testing of Reuterin and Its Impact on the Murine Fecal Microbiome and Volatile Organic Compound Profile
Source: Front Microbiol. 2021 Jul 30;12:699858. doi: 10.3389/fmicb.2021.699858 (PMC8361477; doi:10.3389/fmicb.2021.699858)

Supplement 1: HPLC-chromatogram of 3-HPA in the biotransformation supernatant, detected using a photo diode array. The small peaks are 3-hydroxypropionic acid (3-HP), dimeric 3-HPA and hydrated 3-HPA.

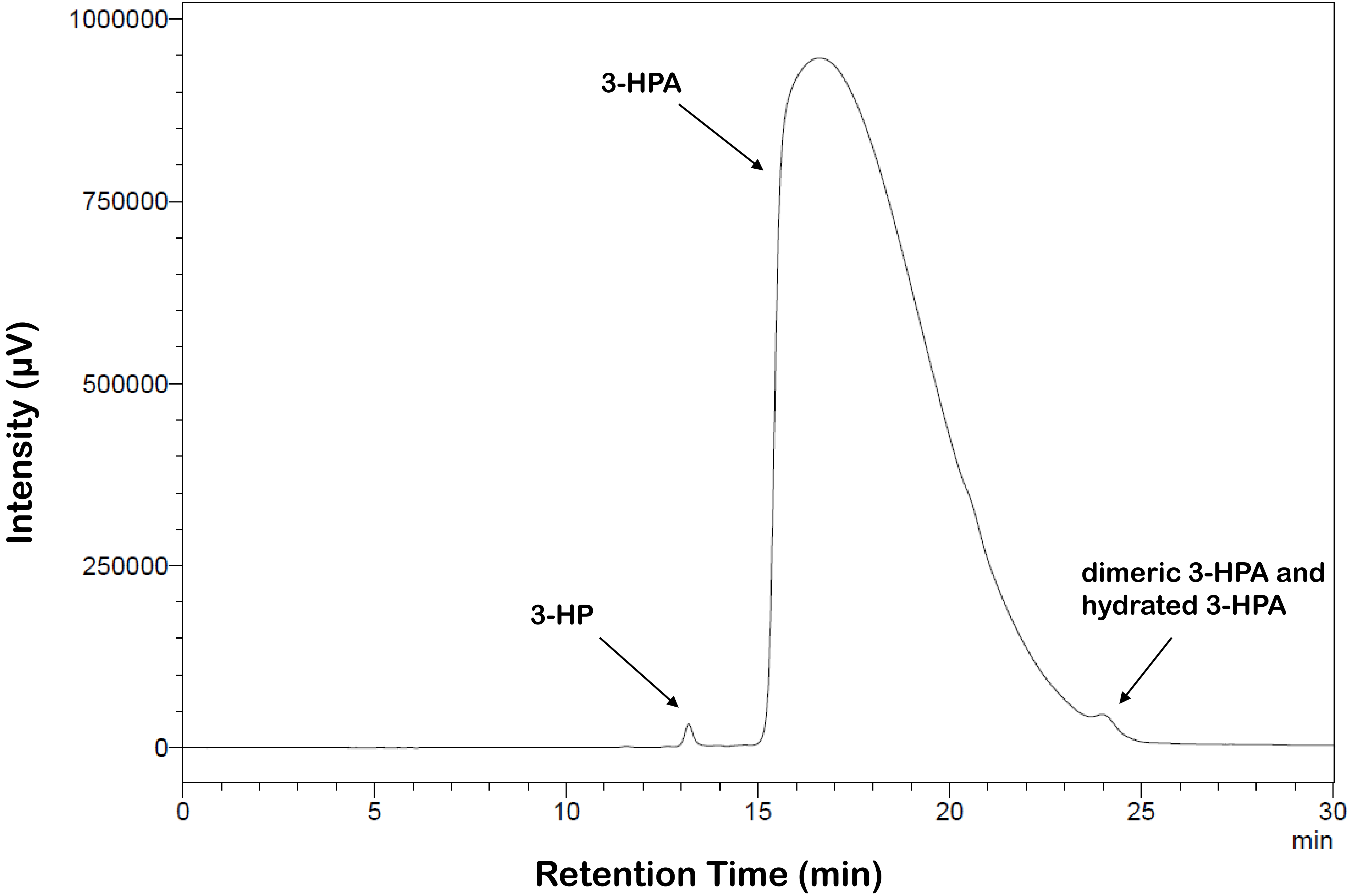

Supplement: Supplementary file 1 [file Image_1.pdf]
