# Supplementary material for: Production, Storage Stability, and Susceptibility Testing of Reuterin and Its Impact on the Murine Fecal Microbiome and Volatile Organic Compound Profile
Source: Front Microbiol. 2021 Jul 30;12:699858. doi: 10.3389/fmicb.2021.699858 (PMC8361477; doi:10.3389/fmicb.2021.699858)
Supplement: Supplementary file 2 [file Table_1.docx]

**Supplement 2:** Alphabetical list of all detected substances in alphabetical order. Std…standard deviation; ME…methyl ester; BE…butyl ester; EE…ethyl ester; PrE…propyl ester; PE…pentyl ester.

| **SUBSTANCE** | **REUTERIN GROUP** | | **REUTERIN MEDIUM GROUP** | | **AMBIENT ROOM AIR** | |
| --- | --- | --- | --- | --- | --- | --- |
|  | mean | std | mean | std | mean | std |
| (E)-2-Butene | 193308 | 146782 | 151908 | 117165 | 0 | 0 |
| (Z)-2-Butene | 105064 | 77610 | 82850 | 78248 | 0 | 0 |
| 2-Butanone | 6967048 | 8149823 | 4389100 | 7880037 | 0 | 0 |
| 2-Heptanone | 835149 | 1971507 | 215089 | 128399 | 0 | 0 |
| 2-Hexanone | 253902 | 237481 | 333556 | 503042 | 0 | 0 |
| 2-Methyl-1-Propene | 91775 | 68269 | 78349 | 74418 | 0 | 0 |
| 2-Methylpropanal | 107618 | 143559 | 104460 | 157625 | 0 | 0 |
| 2-Pentanone | 8652430 | 7662060 | 5417433 | 8364548 | 0 | 0 |
| 2,3-Butandione | 50446043 | 65167037 | 52356580 | 40084669 | 107016 | 17083 |
| 3-Methylbutanal | 2098372 | 2120546 | 1660631 | 4002599 | 0 | 0 |
| Acetacetat-ME | 3099070 | 2162278 | 1715162 | 2001244 | 0 | 0 |
| Acetaldehyd | 2328760 | 2337827 | 1348254 | 1634247 | 20996 | 6605 |
| Acetic Acid | 1019011 | 3222395 | 2738575 | 6595818 | 0 | 0 |
| Acetone | 73558084 | 48461887 | 41866355 | 29941153 | 329028 | 19610 |
| Acidic Acid BE | 1728321 | 2395429 | 564849 | 997247 | 0 | 0 |
| Benzaldehyde | 981215 | 835668 | 1128236 | 1140529 | 57755 | 7250 |
| Butanoic Acid EE | 8506222 | 14168997 | 3002700 | 5176662 | 0 | 0 |
| Butanoic Acid ME | 3346313 | 3472608 | 2116490 | 3119909 | 0 | 0 |
| Butanoic Acid PrE | 2201767 | 4427888 | 1037966 | 1664408 | 0 | 0 |
| Butanoic Acid BE | 784698 | 1643689 | 312779 | 640871 | 0 | 0 |
| Dimethylsulfide | 148823 | 151313 | 182638 | 225256 | 0 | 0 |
| Ethanol | 2133179 | 2764157 | 686357 | 926463 | 0 | 0 |
| Ethylacetate | 7424578 | 8540742 | 3890938 | 6006294 | 0 | 0 |
| Heptane | 35086 | 48650 | 203848 | 290363 | 0 | 0 |
| Hexanal | 197309 | 289220 | 131520 | 143560 | 10790 | 1930 |
| Hexane | 925972 | 2776038 | 124270 | 234279 | 0 | 0 |
| Isoflurane | 24261 | 23819 | 35622 | 18507 | 327616 | 10572 |
| Isopropylalkohol | 0 | 0 | 0 | 0 | 0 | 0 |
| Methylpropionate | 1090986 | 982311 | 1007551 | 1183624 | 0 | 0 |
| Methylvalerate | 487585 | 523426 | 271196 | 220882 | 0 | 0 |
| n-Propylacetate | 2559073 | 2546032 | 2143351 | 2858606 | 0 | 0 |
| o_Xylene | 745800 | 1914043 | 1056060 | 1940572 | 81630 | 18537 |
| Octane | 193949 | 155490 | 295883 | 270364 | 0 | 0 |
| Pentanal | 183037 | 134615 | 178189 | 165708 | 0 | 0 |
| Pentane | 19983 | 32592 | 31017 | 64785 | 0 | 0 |
| Pentane, 3-methyl | 219525 | 694201 | 2995 | 9470 | 0 | 0 |
| Pentane,2-methyl | 134471 | 425236 | 0 | 0 | 0 | 0 |
| Propanal | 2320188 | 2723708 | 754735 | 748316 | 145007 | 66492 |
| Propanol | 0 | 0 | 0 | 0 | 0 | 0 |
| Propen | 83125 | 54750 | 79250 | 65104 | 31339 | 23907 |
| Propionic Acid EE | 2828978 | 3470975 | 1605088 | 2475333 | 0 | 0 |
| Propionic Acid PE | 1058160 | 1083010 | 575043 | 615233 | 0 | 0 |
